# In Memoriam: Professor Laurentiu M. Popescu (1944-2015)

**DOI:** 10.1111/jcmm.12686

**Published:** 2015-09-04

**Authors:** Raymund E Horch

**Affiliations:** Department of Plastic and Hand Surgery, Laboratory for Tissue Engineering and Regenerative Medicine, Speaker of Surgical Departments and Divisions, University Hospital Erlangen, Friedrich Alexander University Erlangen-Nuernberg FAUNuernberg, Erlangen, Germany

**Figure:**
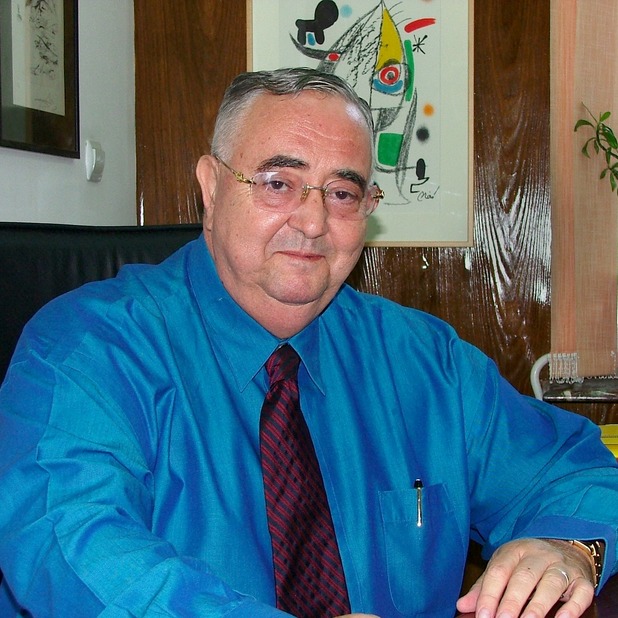

The Remembrance of the good done those we have loved is the only consolation when we have lost them. – Demoustier

Like so many of those who had the chance to learn to know him, I was truly saddened by the all too soon and unexpected death of Laurentiu Mircea Popescu. This outstanding researcher, scientist and teacher influenced many, inspired his environment with his incredible energy, and never seized to follow his ideals to further our comprehension of molecular and cellular medicine.

He was born on April 15th 1944, studied Medicine in Bucharest and then completed a PhD study at the world famous Carol Davila University of Bucharest, the same institution where Insulin had been described for the first time.

It was an opportunity that he readily grasped when he was offered the chance to work as a postdoctoral fellow at the University of Leiden as well as later on as a Fogarty International Fellow at the NIH, Bethesda, Maryland, US. It was his remarkable decision - that was characteristic of him - to finally follow his election and nomination to go back to Bucharest instead of pursuing his undoubtedly promising further career abroad. Back in Bucharest he then chaired the prestigious Victor Babes Institute of Pathology and was a teacher to many young fellows and students.

In retrospect it is of no wonder that during his professional life and his merits he became the Dean of the Medical Faculty and was then elected to preside the whole Carol Davila University, where he took full responsibility for his Alma Mater serving as the rector magnificus (President) from 1992 to 2004.

Since 1995 he became a member and in 2003 the president of the Academy of Medical Sciences Section of the Romanian Academy of Sciences. He then from 2010 to 2011 acted as a vice president and became president of the European Federation of the European Academies of Sciences in 2012. In 2011 he became President-elect of the International Society for Adaptive Medicine.

His scientific oeuvre is best characterized by the discovery of a hitherto undescribed ‘new’ cell type in a time, where, every cell type seemed to have been long since discovered. He realized the enormous potential of these cells for medical research, which has meanwhile also been acknowledged by the scientific community. This did not come by chance, as he himself humbly once reported in the title of one of his seminal papers (Popescu LM, Faussone-Pellegrini MS, TELOCYTES - a case of serendipity: the winding way from Interstitial Cells of Cajal (ICC), via Interstitial Cajal-Like Cells (ICLC) to TELOCYTES, J Cell Mol Med. 2010 Apr;14(4):729–740.). It was the ultimate consequence of tremendous efforts over many years to prove the concept of this new type of cells, from describing caveolar cells and Cajal-like cells until finally characterizing what he then termed ‘Telocytes’.

Together with his fine team of coworkers he characterized Telocytes as an interstitial cell type with a small cell body and extremely long tentacle like extensions, named Telopodes (10–100 μm) and Podomers. These telopodes form a three dimensional network within the surrounding connective tissue. Because Podomers are smaller than 200 nm (and therefore cannot be detected by simple light microscopy) Telocytes had remained undetected for long. Only through the extremely labour intensive, precise multiplanar electron microscopical reconstruction of innumerable histological slices and sections Popescu and his group were able to reveal and decipher the hidden three dimensional cell structure. The fascination of Telocytes lies in many potential functional properties and possible modes of action as well as their relation to putative subepithelial and stromal stem cells. LM Popescu shared all his findings freely with the scientific community and tried to offer what he found with a specially designed website (www.telocytes.com) so that any researcher could (participate in/)benefit from his wisdom. By doing so he stimulated many young and senior researchers from all over the world to join his efforts and to find out more of what Telocytes could be capable of. Possible mechanisms and actions of TC could play a role in angiogenesis, tumorigenesis, Tissue Engineering Horch RE, Beier JP, Kneser U, Arkudas A, Successful human long-term application of in situ bone tissue engineering, J Cell Mol Med. 2014 Jul;18(7):1478–1485, Regenerative Medicine, just to name a few.

Many of his relevant and ground breaking Telocyte papers appeared in the Journal of Cellular and Molecular Medicine (JCMM) which is another proof of his never ending enthusiasm and creativity. When he – some two decades back - started out to found a new journal in this highly competitive scientific field as the Editor in chief many of his friends could not believe that his endeavor could be successful at all. Despite such doubts LM Popescu showed better. It did not take too long for the incredible to become true and JCMM became one of the Top 10 rated journals in its field. He had seen the necessity to bring different areas of research together and his idea was to establish a platform focused on translational medicine, spanning disease-oriented basic research in molecular and cellular biology and pre-clinical investigations into molecular and cellular therapeutics. Even now when we look back it seems almost impossible what LM Popescu and his brilliant team in Romania achieved to manage the work load which a rapidly growing journal will naturally entail. We all thank him for his visionary thoughts and for establishing such an excellent journal that now in his sense truly does provide a unique platform for many highly gifted scientists from everywhere. Above his Telocyte works and other achievements the success story of this scientific ‘baby’ JCMM has shed so much light on the Carol Davila University in Bucharest that he will always be remembered not only in his country but worldwide.

It also came naturally that he was honored with innumerous prizes and accolades, including multiple honorary doctor awards. Among them were highly esteemed awards, such as for instance the Gold medal of the International Academy of Cardiovascular Science. Five of the twenty-five well known scientists who had been honored with this decoration so far later on received the Nobel prize.

My personal relationship with LM Popescu started long ago with his request for a contribution to JCMM in the area of Tissue Engineering and over time I was more than happy to learn to know him personally as an outstanding scientist and impressive personality over time. We eventually became friends and being his guest made one feel like visiting one’s family. Despite his multiple scientific and professional engagements he never forgot his loving family which he regarded as the resting pole and constant source of energy in his personal universe. He was the spiritus rector of many other researchers and I can truly say that he influenced my way of scientifically thinking. Whoever had the opportunity to learn to know LM Popescu can easily imagine what a splendid person we lost. The only consolation that remains is that he will never be forgotten by so many of us. A man’s worth is not measured by ‘how he died’, nor ‘what he gained’, but ‘how he lived’ and ‘what he gave’. Laurentiu Mircea Popescu gave us all so much, that his worth seems immeasurable.

